# Dissertation on the Diseases of the Maxillary Sinus

**Published:** 1843-06-10

**Authors:** 


### BIBLIOGRAPHICAL NOTICES.

*Dissertation on the Diseases of the Maxillary Sinus.* Read before the American Society of Dental Surgeons, at their Third Annual Meeting, held in Boston, July 20, 1842. By Chapin A. Harris, M. D., D. D. S, Professor of Practical Dentistry in the Baltimore College of Dental Surgery, &c. &c. Philadelphia: Lea & Blanchard. 1843. 8vo. pp. 165.

Dr. Harris tells us that in preparing the following memoir he has been "actuated by the belief, that a short, and, at the same time, comprehensive treatise on the morbid affections of this cavity, [the antrum,] would not be altogether unacceptable to the members of the dental profession." No work, devoted exclusively to these diseases, has before been published in our language; and the importance of the subject will be universally admitted, subject, as this structure is, to diseases of the most formidable and dangerous nature. Some of the affections of the antrum are, however, of a more benign character, and yield readily to treatment, especially if instituted in the early stage. But these simple affections frequently become malignant and untractable, if neglected or improperly treated.

"The form which the disease puts on, it must be admitted, is determined by the state of the constitutional health, or some specific tendency of the general system, and we can, therefore, readily imagine, that a cause which, in one person, would give rise to a simple inflammation of the lining membrane, or a mucous engorgement of the sinus, would, in another, produce an ill-conditioned ulcer, fungus hæmatodes, or osteo-sarcoma. But simple inflammation and mucous engorgement of this cavity, not unfrequently cause caries and exfoliations of its surrounding osseous tissues, and as a consequence in some instances, even the destruction of the life of the patient."—(*Introduction*, p. 11.)

Hence the importance of early attention to the disorders of this structure, which are not unfrequently of difficult diagnosis, and which, when much progress is made, if not incurable, become very tedious and troublesome.

"It may, therefore, be safely assumed, that in the very large majority of the cases of disease of the maxillary sinus, the danger to be apprehended results more from neglect than any necessarily fatal character of the malady; so that, in forming a prognosis, the circumstances to be considered should be, the state of the constitutional health, the progress made by the affection, and the nature and extent of the injury inflicted by it upon the surrounding tissues."—(p. 11.)

The author treats of his subject under the following heads: I. Of Inflammation of the Lining Membrane. II. Of a Purulent Condition of its Secretions and Engorgement. III. Of Abscess. IV. Of Ulceration of the Lining Membrane. V. Of Caries, Necrosis, and Softening of its Bony Parietes. VI. Of Tumors of its Lining Membrane and Periosteum. VII. Of Exostoses of its Osseous Parietes. VIII. Of Wounds of its Parietes, and Foreign Bodies in it.

The work itself is evidently the result of much observation and study, and displays considerable research. Indeed, we think that the Dental Profession of the United States has abundant reason to be proud of the position it occupies. An excellent journal of Dental Science is published quarterly at Baltimore, under the auspices of the Society of Dental Surgeons, of which Dr. Harris is one of the Editors. This is combined with a reprint of the best foreign and domestic works on Dentistry. And several recent publications on this department of surgery, by American authors, show much science and skill.

